# Ticks and Tick-Borne Pathogens from Wild Pigs in Northern and Central Florida

**DOI:** 10.3390/insects14070612

**Published:** 2023-07-06

**Authors:** Sarah E. Mays Maestas, Lindsay P. Campbell, Michael P. Milleson, Lawrence E. Reeves, Phillip E. Kaufman, Samantha M. Wisely

**Affiliations:** 1 Entomology and Nematology Department, University of Florida, Gainesville, FL 32608, USA; sarahmays12@gmail.com (S.E.M.M.); lcampbell2@ufl.edu (L.P.C.); lereeves@ufl.edu (L.E.R.); 2 Florida Medical Entomology Laboratory, University of Florida, Vero Beach, FL 32962, USA; 3 National Wildlife Disease Surveillance and Emergency Response Program, United States Department of Agriculture-Animal and Plant Health Inspection Service, Gainesville, FL 32641, USA; michael.p.milleson@usda.gov; 4 Department of Entomology, Texas A&M University, College Station, TX 77845, USA; 5 Department of Wildlife Ecology and Conservation, University of Florida, Gainesville, FL 32608, USA; wisely@ufl.edu

**Keywords:** pathogen surveillance, landscape types, habitat fragmentation

## Abstract

**Simple Summary:**

Ticks are vectors of several agents of human disease, and improvements to traditional surveillance methods are needed to aid in tick-borne disease monitoring and prevention. Invasive wild pigs are broadly distributed in the southern U.S., and removal efforts are often undertaken by local, state, and federal entities. Wild pigs are hosts to several human-biting tick species associated with agents of human disease. This research examines the use of tick collection from wild pigs in the state of Florida as a method of surveillance for ticks and tick-borne pathogens of human concern. Four species of human-biting ticks were collected from wild pigs in this study, yielding similar results to traditional surveillance methods in the state. Known and potential human pathogens were identified in the collected ticks. Landscape features associated with tick diversity and abundance, such as developed spaces, mixed forest, and shrub/scrub habitat were identified, and may be useful for identifying areas of increased risk of encounters with human-biting ticks. These results help to inform tick and tick-borne pathogen surveillance efforts in the state of Florida and suggest that collections from wild pigs may be a useful surveillance tool for continued tick-borne disease surveillance.

**Abstract:**

Invasive wild pigs are distributed across much of the U.S. and are hosts to tick vectors of human disease. Herein, adult ticks were collected from 157 wild pigs in 21 northern and central Florida counties from 2019–2020 during removal efforts by USDA-APHIS Wildlife Services personnel and evaluated for their potential to be used as a method of tick-borne disease surveillance. Collected ticks were identified, screened for pathogens, and the effects of landscape metrics on tick community composition and abundance were investigated. A total of 1415 adult ticks of four species were collected. The diversity of tick species collected from wild pigs was comparable to collections made throughout the state with conventional surveillance methods. All species collected have implications for pathogen transmission to humans and other animals. *Ehrlichia*, *Anaplasma*-like, and *Rickettsia* spp. were detected in ticks collected from wild pigs. These results suggest that tick collection from wild pigs is a suitable means of surveillance for pathogens and vectors. The strongest drivers of variation in tick community composition were the developed open space and mixed forest landcover classes. Fragmented shrub/scrub habitat was associated with increased tick abundance. Similar models may be useful in predicting tick abundance and distribution patterns.

## 1. Introduction

Pathogen surveillance generates data that can be used for outbreak prediction, prevention, and response by observation of the presence, prevalence, and changing distribution of pathogens and patterns of occurrence within populations [1]. The use of domestic or wild animals as surveillance sentinels can improve the efficiency of pathogen detection and play a role in the characterization of emerging diseases [1]. 

Modern sentinel surveillance employs a range of species and techniques. Surveys of seroprevalence, active infection, and mass or unexpected mortality in sentinel species can be used as an indicator of human risk or relative pathogen prevalence. For instance, bird die-offs have historically been used as an early warning signal preceding human cases of West Nile virus [2], and captive chickens are used as sentinel systems for the surveillance of several arboviruses [3]. The sero-surveillance of dogs was identified as an effective indicator of *Borrelia burgdorferi* presence in endemic areas [4,5,6]. Additionally, in areas of recent *Ixodes scapularis* Say range expansion in the U.S., dogs were determined to be effective sentinels of *I. scapularis* presence, while assays of both vector and non-vector tick species collected from dogs was useful in detecting the presence of *B. burgdorferi* [7]. These data suggest that, in addition to acting as sentinels for pathogen detection and surveillance, vertebrates also can be used for the surveillance of pathogen vectors. 

The distributions of many tick species are expanding; the range of *I. scapularis*, the black-legged tick, is expanding both northward and westward [8,9,10], the range of *Amblyomma americanum* (L.), the lone star tick, is expanding northward [10,11], and the range of *Amblyomma maculatum* Koch, the Gulf Coast tick, is expanding northward and eastward [10,12,13,14]. In 2017, *Haemaphysalis longicornis* Neumann, an invasive tick native to eastern Asia, was identified in New Jersey [15]. This tick was subsequently identified in 16 additional states on both domestic and wild animal hosts by the end of 2022 [16]. The collection of ticks from animal hosts may be an efficient method to monitor the spatial and temporal variation of vector tick species and pathogen prevalence, and to recognize the presence of introduced species. 

Swine populations were introduced into what became the U.S. as early as the 1500s by Spanish explorers, and feral populations have been established following repeated introductions in the centuries following [17]. Modern introductions continue, with undomesticated wild Eurasian boar populations released in the 1900s for sport hunting continually being supplemented by free-roaming and escaped domestic pigs; the resulting intermixing feral populations have no natural predators, are highly adaptable, and are rapidly reproducing generalists [18,19]. The current range of wild pigs covers the south and southeastern U.S., California, and parts of several other western, northern and eastern states, with a total of 35 states having counties reporting a wild pig presence by 2021 [20]. 

Wild pigs may be hosts for several tick species, including *A. americanum*, *A. maculatum*, *Dermacentor variabilis* (Say), and *I. scapularis* [21], all of which are human-biting ticks known to transmit a variety of zoonotic pathogens. The use of tick sampling from wild pigs removed from public and private lands may be a useful method of surveillance for ticks and tick-borne pathogens of human concern. 

Habitat types and landscape structures can affect the distribution of tick populations. Ostfeld et al. [22] found that questing *I. scapularis* abundance was greater in forested habitat types compared to shrubland or grassland; however, they found heavy larval infestation of rodents in shrubland and grassland habitats, despite low questing tick abundance. The authors suggest that host habitat use, driven by patch size and connectivity, largely determines the habitat associations of ticks. Some of the species that thrive in edge habitats at forest fragment boundaries, such as the white-footed mouse and white-tailed deer, are primary hosts for tick species such as *I. scapularis* and *A. americanum* [23,24,25]. The abundance of hosts utilizing fragmented habitat edges can result in an increase in tick abundance and an increase in the prevalence of associated pathogens; for example, studies have found positive correlations between *I. scapularis* density and increasing forest fragmentation [26,27]. Knowledge about the habitat variables associated with tick and pathogen abundance can aid in vector management, the prediction of human risk, and vector-borne disease prevention. 

Given the expanding range of multiple tick species and the associated threat of tick-borne disease, improvements to existing surveillance methods are imperative to mitigate the human health risk. The ability to predict and identify areas of increased tick abundance can aid in limiting human exposure to tick vectors. Due to the ecological destruction and the threat of disease transmission to domestic animals associated with wild pigs, removal efforts on the part of federal and state agencies and private landowners are frequently undertaken. The goal of this research was to determine if the collection of ticks from wild pigs can be used as a method to aid in tick and tick-borne pathogen surveillance efforts, and to determine if tick species abundance can be correlated with predictive environmental variables. 

## 2. Materials and Methods

### 2.1. Tick Collection

Tick collection kits were distributed to United States Department of Agriculture Animal and Plant Health Inspection Service (USDA-APHIS) Wildlife Services employees who conducted swine live-trapping and euthanasia operations at approximately 30 trapping locations in the state of Florida. Kits contained a 2 mL BD Vacutainer^®^ EDTA tube (Becton, Dickinson and Company, Franklin Lakes, NJ, USA) for whole blood collection from the swine via cardiac puncture, and two 1.5 mL screw-cap vials containing 95% ethanol for tick collection and preservation. Animal sampling procedures were approved under University of Florida Institutional Animal Care and Use Committee protocol 201910862. 

From August 2019 to September 2020, adult and subadult wild pigs were sampled following euthanasia carried out by USDA-APHIS personnel. Personnel were instructed to collect up to 20 adult ticks per animal from a maximum of 10 pigs per location per season, targeting up to 10 adult ticks from the head and ears, and up to 10 adult ticks from the underline of the body, particularly the regions between the forelegs and the hind legs of the animal. The number of targeted ticks was limited to allow sampling to be completed in a timely manner while processing trapped animals. The GPS coordinates of the capture site were recorded with the collected ticks. Tick and blood samples were stored at −20 °C prior to transportation on ice to the Veterinary Entomology Laboratory at the University of Florida, where they were stored at −80 °C prior to DNA extraction. Samples were assigned to a season based on the sampling date. “Spring” samples were those collected from March–May (49 sampled pigs), “Summer” samples were those collected from June–August (37 sampled pigs), “Autumn” samples were those collected from September–November (41 sampled pigs), and “Winter” samples were those collected from December–February (30 sampled pigs).

### 2.2. Pathogen Screening

The collected ticks were taxonomically identified to life stage, sex, and species, and their engorgement status (flat vs. engorged) was recorded [28,29,30]. Only adult, flat ticks were used for DNA extraction and pathogen screening, to avoid host-blood inhibition of downstream reactions. A Gentra^®^ PureGene^®^ Blood kit (Qiagen, Valencia, CA, USA) was used for DNA extraction of laterally bisected ticks, following an optimized manufacturer protocol [31]. After Proteinase K digestion, the carapace of each tick was removed and placed in 75% ethanol to be held as a voucher specimen. Whole genomic DNA was extracted from whole pig blood using the same extraction kit following an optimized manufacturer-provided protocol. In short, the optimization included increased incubation and centrifugation times; doubling of the volume of the cell lysis solution, protein precipitation solution, and isopropoanol; the addition of Proteinase K (20 mg/mL concentration) for overnight sample incubation at 56 °C in a shaking incubator following the addition of cell lysis solution; and a repeat of the ethanol wash step to wash the DNA pellet.

Eluted samples were used to screen ticks for pathogens, including *Anaplasma*, *Borrelia*, *Ehrlichia*, and *Rickettsia* species. Conventional PCR was performed to screen for *Anaplasma/Ehrlichia* species using primers targeting the *groEL* gene followed by confirmation of the human or mouse strains of *Anaplasma phagocytophilum* positives using primers targeting the *16S* rRNA gene [32,33]. Samples were screened for *Borrelia* species using primers targeting the *Flagellin b* gene [34], and spotted fever group *Rickettsia* species using primers targeting the *ompA* gene [35]. Swine blood samples were screened for infection with *Anaplasma*, *Borrelia*, and *Ehrlichia* using the same primer sets as for the tick screening to investigate the potential role of wild pigs in tick-borne pathogen cycles and to further demonstrate that any detected infections in ticks were representative of infective individuals and not the result of a blood meal from an infected host. Swine samples were not tested for *Rickettsia* infection due to the transovarial transmission patterns of tick-borne *Rickettsia*.

Amplicons from positive samples were submitted for bi-directional Sanger sequencing. Returned sequencing results were visualized and cleaned using GeneStudio™ software contig editor (GeneStudio, Inc., Suwanee, GA, USA). The resulting sequences were compared to sequences published in GenBank using a Basic Local Alignment Search Tool (BLAST) search to determine species identity. Confirmation of specimens identified with a low query coverage when compared to samples in GenBank was achieved by sequencing with an additional groEL primer set [36]. 

### 2.3. Statistical Analyses

To examine the effect of landscape variables on tick community composition, a 1 km radius buffer was generated around the trap location of each sampled animal using ArcMap™ v10.7.1 (ESRI^®^, Redlands, CA, USA). A 1 km buffer size (3.14 km^2^ area) was selected to encompass the likely area of use of wild pigs around the trapping site [17,37,38]. The 2019 National Landcover Raster was downloaded from the Multi-Resolution Land Characteristics Consortium (https://www.mrlc.gov/ Accessed: 12 November 2021). The raster was masked to the state of Florida in ArcMap. Five landcover classes hypothesized to most heavily affect wild pig use and tick abundance were selected for further analysis, and included: developed open space, deciduous forest, mixed forest, shrub/scrub, and herbaceous grassland. The edge density, patch size, and percent landcover of the five selected landcover classes were extracted from each 1 km buffer using the package “landscapemetrics” v1.5.4 [39]. A Pearson’s correlation test was performed in R v1.2.5033 to identify highly correlated variables. Variables with a correlation coefficient < 0.7 and >−0.7 were included in the same models. 

Partial redundancy analysis (pRDA) was performed using the “vegan” Community Ecology package v2.5-7 [40] to examine the effects of the three landscape features with the select landcover classes on the community composition of ticks from wild pigs. Tick count data were transformed using a Hellinger transformation [41]. Here, individual animals served as the site, and tick abundances served as species in the test. The Hellinger-transformed animal-by-tick matrix served as the response variables. Latitude and longitude values provided location information on which to condition each of the models.

Three pRDA models were run, one for each of the landscape features. In the first model, the edge densities of the five landcover classes were the explanatory variables and the Hellinger-transformed animal-by-tick matrix served as the response variables. In the second model, the number of patches of each of the five landcover classes were the explanatory variables and the Hellinger-transformed animal-by-tick matrix served as the response variables. In the third model, the percent landcover of each of the five landcover classes were the explanatory variables, and the Hellinger-transformed animal-by-tick matrix served as the response variables. A permutated ANOVA with 999 iterations was used to test the significance of explanatory variables. 

We used generalized linear mixed effects models (GLMMs) to further investigate the effects of selected landscape variables and habitat types on *A. americanum* abundances. Pairs plots were generated to observe tick counts with each landcover variable to identify the presence of outliers and non-linearity. Because many landscape metrics are known to be highly collinear, variance inflation factor (VIF) values were calculated across all variables to test for collinearity in the dataset. High values were exhibited across the majority of variables. Thus, variables were divided into three candidate sets representing each of the landscape metrics investigated in the models (i.e., number of patches, edge density, and percent land cover) and the VIF calculations were repeated to ensure that multicollinearity was not present in environmental variables within each candidate set using a threshold VIF value < 3.0.

Visual inspection of the distribution of tick abundances revealed a large proportion of zeros and a wide distribution of counts along the x-axis, indicating strong potential for overdispersion [42]. In order to quantify the effects of edge density, number of patches, and percent landcover on *A. americanum* abundance, data were transformed using Log(x + 1), and fit to a GLMM with a Gaussian distribution including a pig-by-site random effect using the ‘glmmTMB’ package in R [43]. Models including all combinations of variables within each candidate set were generated using the “dredge” function in the ‘MuMIn’ package in R [44]. Results were evaluated using an information criterion approach, ranking models from lowest to highest Akaike’s Information Criterion (AIC) scores, with the lower AIC scores indicating better-supported models [45]. In addition, AIC weights (AICw) were calculated for each model, providing information about the weight of evidence supporting the model, with higher values indicating stronger support. We used a threshold of delta AIC < 2 to identify the number of models contributing information. Effect curves for the most parsimonious model from each candidate set were generated with the ‘ggeffects’ package in R used to visualize the strength of the variable with 95% confidence intervals [46]. Model diagnostics of the most parsimonious model from each candidate set were performed using the ‘Dharma’ package in R [47], and model summaries include information about relevant predictors, those with CIs that did not cross zero, and *p*-values < 0.05. Tests for residual spatial autocorrelation were performed using a spatial correlogram in the ‘ncf’ package in R [48].

## 3. Results

### 3.1. Tick Collection

**Figure 1:**
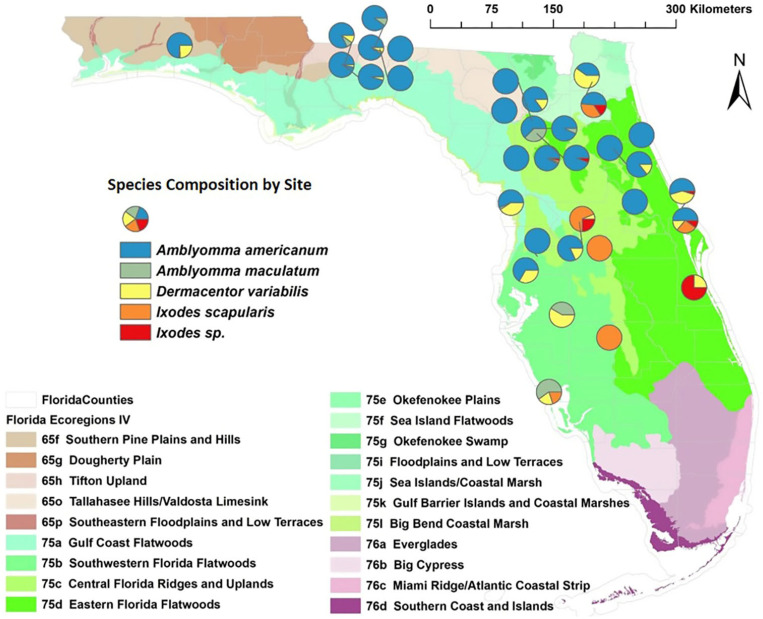
Tick species composition and distribution of sampling locations where collections were made from wild pigs (2020–2021) across the state of Florida. Up to 20 ticks were removed from each sampled individual. A total of 1415 ticks were collected from 157 wild pigs in 80 trap sites at 30 locations across northern and central Florida. The tick species composition is displayed by collection site and ecoregion, when collections were made from multiple ecoregions within one trapping site.

A total of 1415 adult ticks were collected from 157 adult and sub-adult wild pigs captured in 80 individual traps at 31 trapping locations in 21 northern and central Florida counties: 1117 *A. americanum*, 32 *A. maculatum*, 149 *D. variabilis*, 79 *I. scapularis*, and 38 damaged specimens identified as *Ixodes* sp. (Table 1, Figure 1). The damaged specimens were presumed to be *Ixodes scapularis* but were considered separately in further data analysis. 

The greatest numbers of ticks were collected in the spring and summer (Table 1). *Amblyomma americanum* and *D. variabilis* were most frequently encountered in the spring and summer, *A. maculatum* in the autumn, and *I. scapularis* in the autumn and winter (Table 1). *Ixodes scapularis* and *Ixodes species* were more frequently encountered in the central portion of the state (Figure 1). *Amblyomma maculatum* was the least frequently encountered tick species but was distributed throughout the northern and central parts of the state (Figure 1). Although *A. americanum* were the most frequently encountered ticks overall, they were absent at the southernmost collection sites (Figure 1). 

### 3.2. Pathogen Screening

A total of 856 ticks from 20 counties (all unfed ticks collected) were screened for pathogens: 714 *A. americanum*, 18 *A. maculatum*, 75 *D. variabilis*, 37 *I. scapularis*, and 12 *Ixodes* sp. No ticks were infected with *Borrelia* species. There was a 3.3% infection prevalence of *Ehrlichia* and *Anaplasma*-like species (28/856) distributed among 19 *A. americanum*, 1 *D. variabilis*, 7 *I. scapularis* and one *Ixodes* sp. In total, 14 ticks were infected with *E. ewingii*: 13 *A. americanum*, >99% homologous to *E. ewingii* previously isolated from *A. americanum* (GenBank KJ907744), and one *D. variabilis*; the sequence quality of this amplicon was poor and was only 83.8% homologous to GenBank sequence KJ907744. Four *A. americanum* were infected with *E. chaffeensis*, >99% homologous to *E. chaffeensis* previously isolated from *A. americanum* (GenBank KJ907753), and two *A. americanum* were infected with *Ehrlichia* sp. Panola Mountain that was >99% similar to a previous isolate from *A. americanum* (GenBank KJ907753). 

Seven *I. scapularis* and one *Ixodes* sp. were infected with a bacterial species identified with the *groEL* gene as *A. phagocytophilum*-like, having a low-percentage match to *A. phagocytophilum* (75.1–78.6% homologous to *A. phagocytophilum* GenBank MH722254); however, these samples were not amplified with the *16S* primers targeting *A. phagocytophilum*. All eight samples were a >97% match to *Candidatus* Cryptoplasma californiense, identified from *Ixodes pacificus* Cooley and Kohls in California (GenBank KP276602), although the total query coverage of the sequences in GenBank was only 64%. The eight samples were subsequently amplified and sequenced using primers from Eshoo et al. [36] targeting the *groEL* gene. This increased the query cover to >95%, with six of the eight samples having >97% homology to *Candidatus* Cryptoplasma californiense identified from *I. pacificus* in California (GenBank KP276601). The remaining two of the eight samples were 87% and 91%, respectively, homologous to GenBank KP276601; these samples appeared to be co-infected, with sequences having multiple peaks. 

There was a 44.7% *Rickettsia* prevalence overall (383/856) in the sampled ticks. There was a 48.3% *Rickettsia* infection prevalence in *A. americanum* (345/714), while the prevalence in *A. maculatum* was 11.1% (2/18). The *Rickettsia* infection prevalence in *D. variabilis* was 5.3% (4/75). The *Rickettsia* infection prevalence in *I. scapularis* was 73% (27/37), and in *Ixodes* sp. it was 41.7% (5/12). 

Of the 383 *Rickettsia*-positive samples, 155 were sequenced. *Rickettsia amblyommatis* was detected in 137 *A. americanum* samples, 135 of which were >99% similar to *R. amblyommatis* amplicon sequences previously detected in an *A. americanum* collected in north-central Florida (GenBank MN313363). The two remaining *A. americanum* samples returned sequences that were a 97–98% match to the same isolate. One *A. maculatum* was infected with *R. amblyommatis*, with a sequence that was 98% similar to an *R. amblyommatis* isolate from *A. americanum* in Texas (GenBank MN336348). One *A. maculatum* was infected with *R. parkeri* with a sequence that was 100% similar to an *R. parkeri* isolate from *A. maculatum* in Texas (GenBank KP861344). One *D. variabilis* was infected with *R. amblyommatis* with a sequence that was 100% similar to GenBank MN313363. Three additional *Rickettsia*-positive *D. variabilis* samples that were collected from the same host animal as the sequenced sample were not sequenced. Ten *I. scapularis* and five *Ixodes* sp. were infected with a rickettsial endosymbiont that was ≥99% similar to an *I. scapularis* rickettsial endosymbiont detected in an *I. scapularis* collected in North Carolina (GenBank KP172259). 

**Figure 2:**
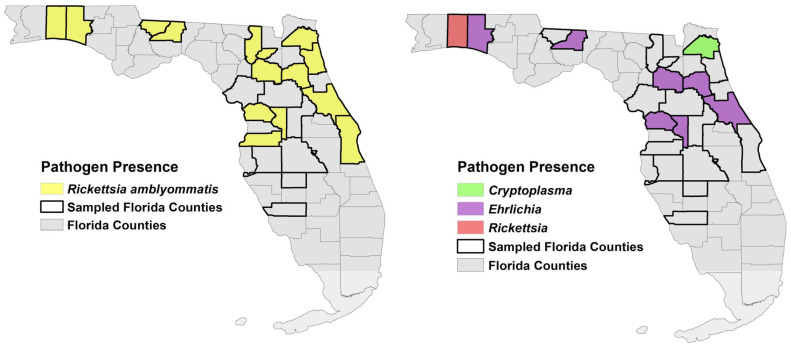
Distribution of *Rickettsia amblyommatis* (**left** panel) and other known or potential pathogens (**right** panel) in ticks collected from wild pigs in northern and central Florida (2020–2021) by county. Sampled counties are indicated by black county boundaries on the maps. The presence of pathogens within sampled counties is indicated by color representing a pathogen genera or species.

Bacteria species detected in ticks were distributed across collection sites in northern and central Florida but were not detected at the southernmost collection sites (Figure 2). No infection with *Anaplasma*, *Borrelia*, or *Ehrlichia* was detected in any swine blood samples (*n* = 157) from these sites.

### 3.3. Statistical Analyses

**Figure 3:**
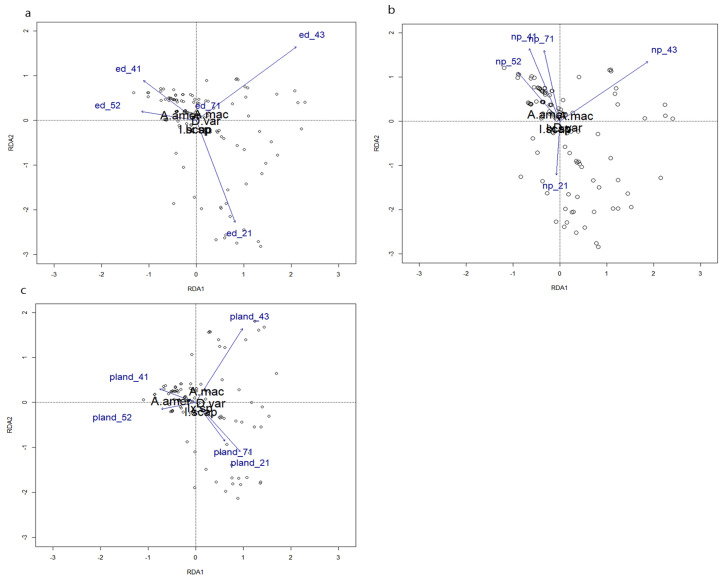
Tri-plots of tick species collected from wild pigs with three landscape metrics of five landcover classes as explanatory variables within a 1 km buffer. Tick species A.amer = *Amblyomma americanum*; A.mac = *Amblyomma maculatum*; D.var = *Dermacentor variabilis*; I.scap = *Ixodes scapularis*; and Ix.sp = *Ixodes* sp. Landcover classes 21 = Developed open space; 41 = Deciduous forest; 43 = Mixed forest; 52 = Shrub/Scrub; 71 = Herbaceous grassland. Triplots of weighted averages, scaling = 2. (**a**) Edge density; (**b**) number of patches; (**c**) percent landcover. Ticks were collected from 157 animals at 31 sites (2020–2021).

The results of the Pearson’s correlation test indicated that there was little correlation among habitat types within each landscape feature (edge density, patch number, and percent landcover). The adjusted R^2^ values of the proportion of constrained variance in the pRDAs in which tick community composition was the response variable and edge density, number of patches, and percent landcover of the five selected habitat types where the explanatory variables were 2.9%, 2.5%, and 5.5%, respectively. The proportion of variance conditioned on the X and Y coordinates of trap location was 12.8% in all three models (Table 2). The models for edge density, patch number, and percent landcover were all significant (*p* = 0.014, *p* = 0.021, and *p* < 0.001, respectively). ANOVA results for the individual explanatory variables indicated that the edge density of developed open space (F_1,149_ = 3.03, *p* = 0.023) and mixed forest (F_1,149_ = 4.13, *p* = 0.007), the number of patches of mixed forest (F_1,149_ = 4.63, *p* = 0.008), and the percent landcover of developed open space (F_1,149_ = 4.88, *p* = 0.004), mixed forest (F_1,149_ = 5.70, *p* = 0.003), and herbaceous grassland (F_1,149_ = 3.06, *p* = 0.026) explained a significant proportion of the variance in tick species communities (Figure 3). When edge density was examined, developed open space and mixed forest were the drivers of variation in tick community composition; there was a weak association between *A. americanum* and deciduous forest and shrub/scrub, and a weak association between *A. maculatum* and herbaceous grassland and deciduous forest. *Dermacentor variabilis* showed no strong correlation with any habitat type, and *I. scapularis* and *Ixodes* sp. were more correlated with open developed space (Figure 3a). Similar patterns were evident when examining patch number (Figure 3b). When the percent landcover of each habitat type was examined, developed open space, mixed forest, and herbaceous grassland were the most important drivers of variation in tick community composition. There were weak associations between *A. americanum* and deciduous forest and shrub/scrub; between *A. maculatum* and mixed forest; and between *D. variabilis*, *I. scapularis*, and *Ixodes* sp. and herbaceous grassland and developed open space (Figure 3c). 

When examining the relationship between *A. americanum* abundance and individual landscape metrics, the residual diagnostics showed slight non-linearity, but this factor was not strong enough to generate significant nonparametric dispersion. Variance inflation factor (VIF) values were low between all variables in each of the three candidate sets, indicating that there was little multicollinearity between the selected landcover classes within each landscape metric (Supplemental Material Table S1). Models of edge density and percent landcover of the selected landcover classes had the lowest AIC scores, while models of patch number had the highest AIC values (Table 3). Shrub/scrub and herbaceous grassland landcover classes were consistent variables in the best fit models.

**Figure 4:**
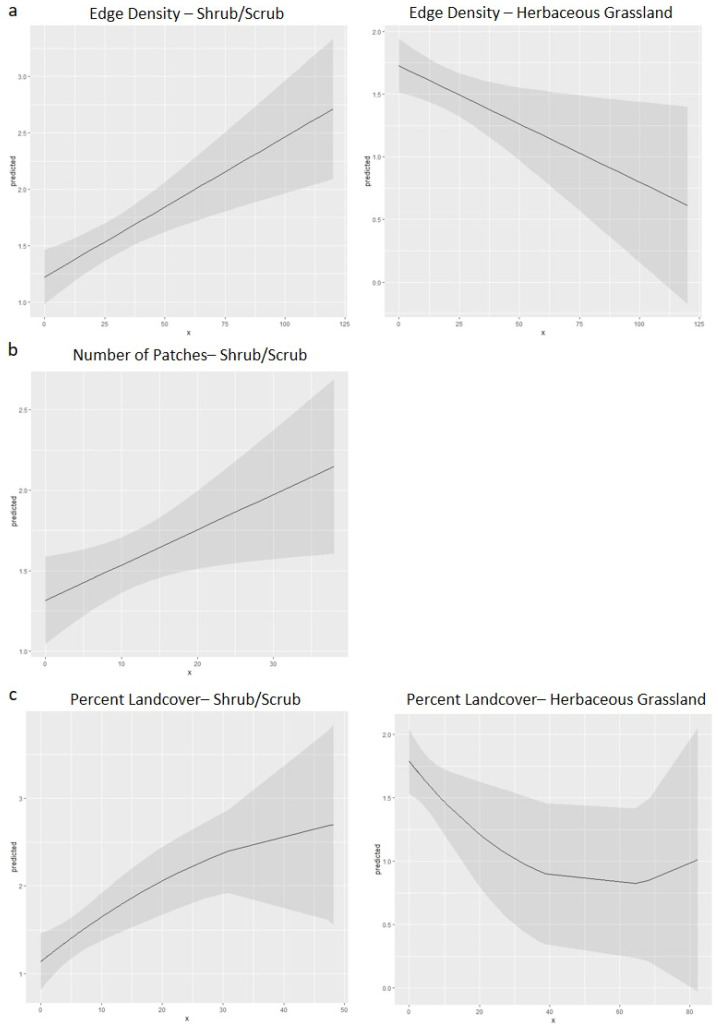
Effect curves with 95% confidence intervals for each variable in the most parsimonious model of each landscape metric used to predict abundance of *Amblyomma americanum* collected from wild pigs in north and central Florida: (**a**) two variables in the edge density model; (**b**) one variable in the number of patches model; and (**c**) two variables in the percent landcover model.

Results for the edge density of the select landcover classes within a 1 km buffer distance as a predictor of *A. americanum* abundance on wild pigs indicated that five models were included in the best set of models, defined as models that do not exceed a threshold of ∆AIC < 2 (Table 3, Supplemental Material Table S2). In the best set of models of edge density, all five models included shrub/scrub and herbaceous grassland landcover classes as variables, followed by mixed forest and developed open space. Parameter estimates indicated a negative effect of herbaceous grassland, and a positive effect of shrub/scrub, mixed forest, and developed open space on *A. americanum* abundance. Deciduous forest was included as a variable in only one model, where parameter estimates indicated a negative effect on *A. americanum* abundance. The most parsimonious model had an AIC weight (AIC_w_) of 0.205 and included edge density of shrub/scrub and herbaceous grassland as variables in the model. Both variables were significant (Table 4). The effect curves for the edge density of shrub/scrub and herbaceous grassland indicated that, as values of edge density of shrub/scrub landcover increased, *A. americanum* abundance was predicted to increase; as values of herbaceous grassland increased, *A. americanum* abundance on wild pigs was predicted to decrease (Figure 4a). 

The results for the number of patches of the select landcover classes measured within a 1 km buffer distance as a predictor of *A. americanum* abundance on wild pigs indicated that nine models were included in the best set of models (Table 3, Supplemental Material Table S2). All landscape classes appeared as variables multiple times within these nine models, although shrub/scrub was the most frequently included variable, followed by mixed forest. Parameter estimates indicated a positive effect of both variables on *A. americanum* abundance. The most parsimonious model had an AIC_w_ of 0.106 and included only number of patches of shrub/scrub as a variable in the model, and this variable was significant (Table 4). The effect curve for the number of patches of shrub/scrub landcover indicated that as the number of patches of shrub/scrub landcover increased, *A. americanum* abundance on wild pigs was predicted to increase (Figure 4b). 

The results for the percent landcover of the select landcover classes measured within a 1 km buffer distance as a predictor of *A. americanum* abundance on wild pigs indicated that three models were included in the best set of models (Table 3, Supplemental Material Table S2). The percent landcover of shrub/scrub and herbaceous grassland were included as variables in all three models. Percent landcover of mixed forest and of developed open space were each included as an additional variable in one of the three models. Parameter estimates indicated a positive effect of the percent landcover of shrub/scrub on *A. americanum* abundance, and a negative effect of the percent landcover of herbaceous grassland on *A. americanum* abundance. The most parsimonious model included percent landcover of shrub/scrub and percent landcover of herbaceous grassland as variables in the model. A polynomial term was added to the model covariates to improve the model fit, wherein shrub/scrub was a significant variable, while herbaceous grassland was marginally significant (Table 4). The effect curves for the percent landcover of shrub/scrub and herbaceous grassland indicated that, as the percent of shrub/scrub landcover increased, *A. americanum* abundance was predicted to increase, and that as the percent landcover of herbaceous grassland increased, *A. americanum* abundance was predicted to decrease (Figure 4c). 

The model diagnostics indicated a good model fit for all models described (Supplemental Material Figure S1). Tests for residual spatial autocorrelation were performed using a spatial spline correlogram in the ‘ncf’ package in R. The results indicated that significant autocorrelation was not present in the most parsimonious model of each of the landscape metrics (Supplemental Material Figure S2).

## 4. Discussion

The diversity of tick species collected from the wild pigs was comparable to prior collections made throughout the state with conventional surveillance methods such as dragging and flagging [49,50], and sampling from animals [51,52,53,54]. All the tick species collected have implications for zoonotic pathogen transmission. Thus, collections from wild pigs can be a useful method to aid in statewide surveillance for tick pathogen vectors. While the species of ticks detected in this study are comparable to other large-scale surveys in the state, the relative abundances of each of the detected species differed from some studies. Proportionately more *A. maculatum* and *D. variabilis* were collected from wild pigs in this study than from surveys of questing ticks in the state, while proportionately fewer *I. scapularis* were detected [49,50]. Allan et al. [51] reported that *I. scapularis* was the most common tick they collected from wild pigs, followed by *A. americanum*. Their sampling, however, was carried out exclusively during the autumn deer hunting season, which coincides with the peak activity of adult *I. scapularis* and could account for the increased abundance of *I. scapularis*. Hertz et al. [53] sampled a variety of wildlife host species but detected similar relative abundances of the four species detected herein; Hertz et al. also identified small numbers of two species (*Ixodes affinis* Neumann and *Ixodes texanus* Banks) that were not detected from the swine herein. A southern Florida study that collected ticks from wild pigs, and by dragging on the same property, detected four species of adult ticks from wild pigs and only three species from drags, although immature-stage ticks were more frequently collected on drags [54]. Notably, tick species that are less frequently collected by dragging/flagging, such as *A. maculatum* and *D. variabilis*, may be encountered more frequently when sampling from host animals; therefore, animal sampling may be a useful method for monitoring the presence of ticks that are underrepresented by other collection methods. 

Although immature ticks may be less frequently detected on wild pigs, adult ticks, which have completed multiple blood meals, are often the preferred stage for general pathogen surveillance due to transstadial accumulation of pathogens. In this study, collectors were instructed to collect only adult ticks to maximize sampling efforts. 

Three pathogenic species of *Ehrlichia* were detected in ticks collected from wild pigs. *Ehrlichia chaffeensis*, *E. ewingii*, and *Ehrlichia* sp. Panola Mountain each have been previously detected, typically at low prevalence, in questing and host-collected ticks in Florida [49,55,56,57]. The overall prevalence of *E. chaffeensis* in this study was 0.47%, the prevalence of *E. ewingii* was 1.6%, and the prevalence of *Ehrlichia* sp. Panola Mountain was 0.23%. The 20 ticks infected with pathogenic *Ehrlichia* came from 14 animals at nine locations in seven counties (Figure 2). The *Ehrlichia* prevalence detected herein is similar to most detections in prior studies from both questing and host-collected ticks. This indicates that tick collections from wild pigs as a surveillance method for vector-borne pathogens yields similar estimates of pathogen prevalence as dragging and flagging. 

Seven *I. scapularis* and one *Ixodes* sp. were infected with an *A. phagocytophilum*-like organism, *Candidatus* Cryptoplasma californiense, that was originally identified in *I. pacificus* in California. In their initial description of this organism, Eshoo et al. [36] noted the detection of isolates with high similarity to their California isolates from studies in eastern Asia, Europe, and northern Africa, and suggested that this organism may be widespread in *Ixodes*. The potential for this *Cryptoplasma* species to cause illness in humans or other animals is unknown. Five of the eight infected ticks were removed from the same animal. The other three infected ticks came from three different animals. All four animals were trapped at the same collection site in the northeastern corner of the state (Figure 2). To our knowledge, this was the first detection of this organism in ticks in Florida.

The high prevalence of *Rickettsia* in ticks collected from wild pigs was driven by the 73% prevalence of rickettsial endosymbionts in *Ixodes* ticks and the 48% prevalence of *Rickettsia* species in *A. americanum*. All sequenced amplicons from *A. americanum* were identified as *R. amblyommatis*. Infection with *R. amblyommatis* also was detected in one *D. variabilis* and one *A. maculatum*. It is notable that *R. parkeri*-infection was detected in only one of 18 *A. maculatum* samples, as a high prevalence of Rickettsia spp. (~30%), primarily identified as *R. parkeri* in *A. maculatum* from Florida populations, has previously been reported [56]. With the exception of *R. parkeri*, the *Rickettsia* species detected in this study were either non-pathogenic or of questionable pathogenicity [57]. 

Landscape fragmentation and the availability of edge habitat was shown to be an important driver of the presence of various tick species, likely due to the close association between several tick species and host species that utilize edge habitat [22,23,24,25,26,27]. In this study, the redundancy analysis suggested that the edge density and patch number of mixed forest and developed open space, and the percent landcover of mixed forest, developed open space, and grassland landcover were the major drivers of variation in tick community composition (Figure 3). The collected tick species were most closely associated with deciduous forest, shrub/scrub, herbaceous grassland and open developed space. As landscape fragmentation increases, tick community composition is also expected to be affected. 

The data collected in this study were used to produce GLMMs to determine which landscape metrics were most strongly correlated with the abundance of *A. americanum*, the most commonly encountered tick in the study. Two of the utilized metrics, edge density and number of patches of selected landcover classes, served as indicators of increased fragmentation. The most parsimonious models within each landscape metric of *A. americanum* abundance always included the shrub/scrub landcover class (Table 3, Supplemental Material Table S2). As the edge density, number of patches, or percent landcover of the shrub/scrub landcover class increased, the predicted values of *A. americanum* abundance increased as well. The most parsimonious model for two of the three landscape metrics also included the herbaceous grassland landcover type as a covariable. As the edge density or percent landcover of herbaceous grassland increased, the predicted values for *A. americanum* abundance decreased. 

The strongest models generated herein with the lowest AIC values and highest AIC_w_, or relative likelihood, were the model of edge density where shrub/scrub and herbaceous grassland were included as covariables, and the model of percent landcover where shrub/scrub and herbaceous grassland as covariables. These results demonstrate that these two landcover types are important drivers of tick abundance. The herbaceous grassland landcover likely has a direct negative effect on *A. americanum* abundance, while species such as *A. maculatum* seem to thrive in grassland habitat [58,59]. The positive effect of shrub/scrub habitat may be the result of host use and movement patterns, such as the use of the shrub/scrub habitat by wild pigs as they move between areas of resource utilization (i.e., foraging near the edges of pasture, cropland, or grassland, but sheltering in more wooded areas) and utilization by generalist species such as white-tailed deer, another primary host of *A. americanum* [25]. A Florida study that evaluated environmental associations to predict *A. americanum* distribution found that their forest variable was among those predicted to most heavily influence *A. americanum* presence [60]. However, this study surveyed questing ticks. Other studies have indicated that the abundance of ticks on a host in an area may be high even when questing tick numbers are low due to host movement patterns [22]. 

The collection of four human-biting tick species from wild pigs suggest that this host sampling method is promising for human tick-borne pathogen and vector surveillance. Our sampling of wild pigs resulted in the collection of a greater abundance of tick species that can be difficult to detect with traditional sampling methods such as flagging/dragging; thus, this surveillance method may be useful in detecting the presence or monitoring the range expansion of species that may otherwise be difficult to detect. The identification of human pathogens in ticks collected from wild pigs, and the detection of a novel tick-borne bacteria in Florida from wild pig-collected ticks further demonstrate the usefulness of wild pigs as a method of surveillance for ticks and tick-borne pathogens. The integration of large-scale sampling from wild pigs with other surveillance methods can augment and improve current vector-borne disease surveillance efforts. 

## Figures and Tables

**Table 1:** Tick species totals and collections by season from wild pigs captured in northern and central Florida from August 2020 through September 2021.

Species	Total	Spring	Summer	Autumn	Winter
*Amblyomma americanum*	1117	436	453	60	168
*Amblyomma maculatum*	32	2	5	25	0
*Dermacentor variabilis*	149	80	41	19	9
*Ixodes scapularis*	79	0	0	64	15
*Ixodes* sp.	38	1	0	28	9
Total ticks	1415	519	499	196	201
Total pigs sampled	157	49	37	41	30

**Table 2:** Partitioning of variance of pRDA models of tick community composition on wild pigs in northern and central Florida with edge density, number of patches, and percent landcover of developed open space, deciduous forest, mixed forest, shrub/scrub, and herbaceous grassland within a 1 km buffer as explanatory variables.

Partial RDA—Partitioning of Variance						
	Edge Density 1 km		Number of Patches 1 km		Percent Landcover 1 km	
	Inertia	Proportion	Inertia	Proportion	Inertia	Proportion
Conditioned	0.056	0.128	0.056	0.128	0.056	0.128
Constrained	0.025	0.056	0.022	0.052	0.035	0.081
Unconstrained	0.354	0.815	0.356	0.819	0.343	0.791
	F = 2.06, df = 5149, *p* = 0.014 Adjusted R^2^ = 0.029		F = 1.90, df = 5149, *p* = 0.021 Adjusted R^2^ = 0.025		F = 3.05, df = 5149, *p* < 0.001 Adjusted R^2^ = 0.055	

**Table 3:** Akaike Information Criterion (AIC) table presenting the best models to predict the abundance of *Amblyomma americanum* collected from wild pigs in northern and central Florida (2020–2021). Landcover classes were the predictor variables in the model.

Landscape Metric	Intercept	Dev. Open Space	Decid. Forest	Mixed Forest	Shrub/Scrub	Grassland	df	Log(*Lᵢ*) *	AIC	∆AIC ^†^	AIC_w_^‡^
Edge Density	1.391	X ^§^	X	X	0.012437	−0.00929	6	−231.695	475.389	0.000	0.205
Percent Landcover	1.424	X	X	X	0.034478	−0.01278	6	−231.714	475.428	0.000	0.325
Edge Density	1.289	X	X	0.007797	0.013215	−0.00883	7	−230.919	475.839	0.450	0.164
Edge Density	1.181	0.003159	X	0.009587	0.013057	−0.00833	8	−230.223	476.446	1.057	0.121
Edge Density	1.331	0.002213	X	X	0.012201	−0.00901	7	−231.337	476.674	1.285	0.108
Percent Landcover	1.388	0.007351	X	X	0.034619	−0.01269	7	−231.526	477.052	1.624	0.144
Edge Density	1.392	X	−0.00218	X	0.012857	−0.00935	7	−231.661	477.321	1.932	0.078
Percent Landcover	1.406	X	X	0.010425	0.034777	−0.01258	7	−231.678	477.357	1.928	0.124
Patch Number	1.316	NA	NA	NA	0.021892	NA	5	−236.504	483.008	0.000	0.106
Patch Number	1.465	X	0.019994	X	X	X	5	−236.650	483.301	0.293	0.092
Landscape Metric	Intercept	Dev. Open Space	Decid. Forest	Mixed Forest	Shrub/Scrub	Grassland	df	Log(*Lᵢ*)	AIC	∆AIC	AIC_w_
Patch Number	1.111	0.009235	X	0.023128	0.025415	−0.00999	8	−233.907	483.815	0.807	0.071
Patch Number	1.247	0.005662	X	X	0.021306	X	6	−235.920	483.840	0.831	0.070
Patch Number	1.114	0.009175	0.008612	0.018955	0.016897	X	8	−233.990	483.979	0.971	0.065
Patch Number	1.279	0.0095	0.017761	0.014107	X	X	7	−235.089	484.179	1.170	0.059
Patch Number	1.346	X	X	X	0.026004	−0.01029	6	−236.131	484.262	1.254	0.057
Patch Number	1.262	X	X	0.015632	0.026143	−0.00981	7	−235.300	484.599	1.591	0.048
Patch Number	1.265	X	0.008696	0.011459	0.017647	X	7	−235.363	484.725	1.717	0.045

* Log(*Lᵢ*) is the log likelihood, ^†^ ∆AIC is the change in AIC values compared to the most parsimonious model within each landscape metric. ^‡^ AICw is the AIC weight, or the conditional probability of the model. ^§^ Values of X under a variable column for a specific model row indicates that the variable was not included in the model.

**Table 4:** Summary table of the most parsimonious models for each of three landscape metrics to predict the abundance of *Amblyomma americanum* on wild pigs sampled in northern and central Florida (2020–2021).

Model Type and Variables	Estimate	Std. Error	Z-Value	*p*-Value
Edge Density				
Intercept	1.391	0.123	11.308	<0.001
Shrub/Scrub	0.012	0.003	3.779	<0.001
Herbaceous grassland	−0.009	0.004	−2.403	0.016
Number of Patches				
Intercept	1.316	0.138	9.526	<0.001
Shrub/Scrub	0.022	0.010	2.248	0.025
Percent Landcover				
Intercept	1.417	0.126	11.223	<0.001
Shrub/Scrub	0.056	0.025	2.225	0.024
I Shrub/Scrub^2^	−0.000	0.000	−0.786	0.432
Herbaceous grassland	−0.035	0.019	−1.952	0.051
I Herbaceous grassland^2^	0.000	0.000	1.230	0.219

## Data Availability

Data are available upon request to the corresponding author. Data are not publicly available due to privacy restrictions.

## Supplementary Materials

The following supporting information can be downloaded at: https://www.mdpi.com/article/10.3390/insects14070612/s1, Figure S1: QQ plots, residual diagnostics, and dispersion tests for model fit of most parsimonious model of each candidate set used to predict abundance of *Amblyomma americanum* collected from 157 wild pigs in north and central Florida. (a) Edge density, (b) Number of patches, (c) Percent landcover; Figure S2: Spatial correlogram plot with 95% confidence intervals to test for residual spatial autocorrelation in each of the three landscape variables used to predict abundance of *A. americanum* collected from wild pigs in north and central Florida. 1000 iterations were used to generate a bootstrap distribution; Table S1: Variance Inflation Factor (VIF) values for each candidate set of variables within each landscape metric for the prediction of the abundance of *A. americanum* from wild pigs in northern and central Florida; Table S2: Akaike Information Criterion (AIC) table of models of three landscape metrics where selected landcover classes are predictor variables to predict the abundance of *A. americanum* collected from wild pigs in north and central Florida (2020–2021).
