# Supplementary material for: Ticks and Tick-Borne Pathogens from Wild Pigs in Northern and Central Florida
Source: Insects. 2023 Jul 6;14(7):612. doi: 10.3390/insects14070612 (PMC10380241; doi:10.3390/insects14070612)
Supplement: Supplementary file 1 [file insects-14-00612-s001.zip › insects-2468975-supplementary.pdf]

Supplementary Material

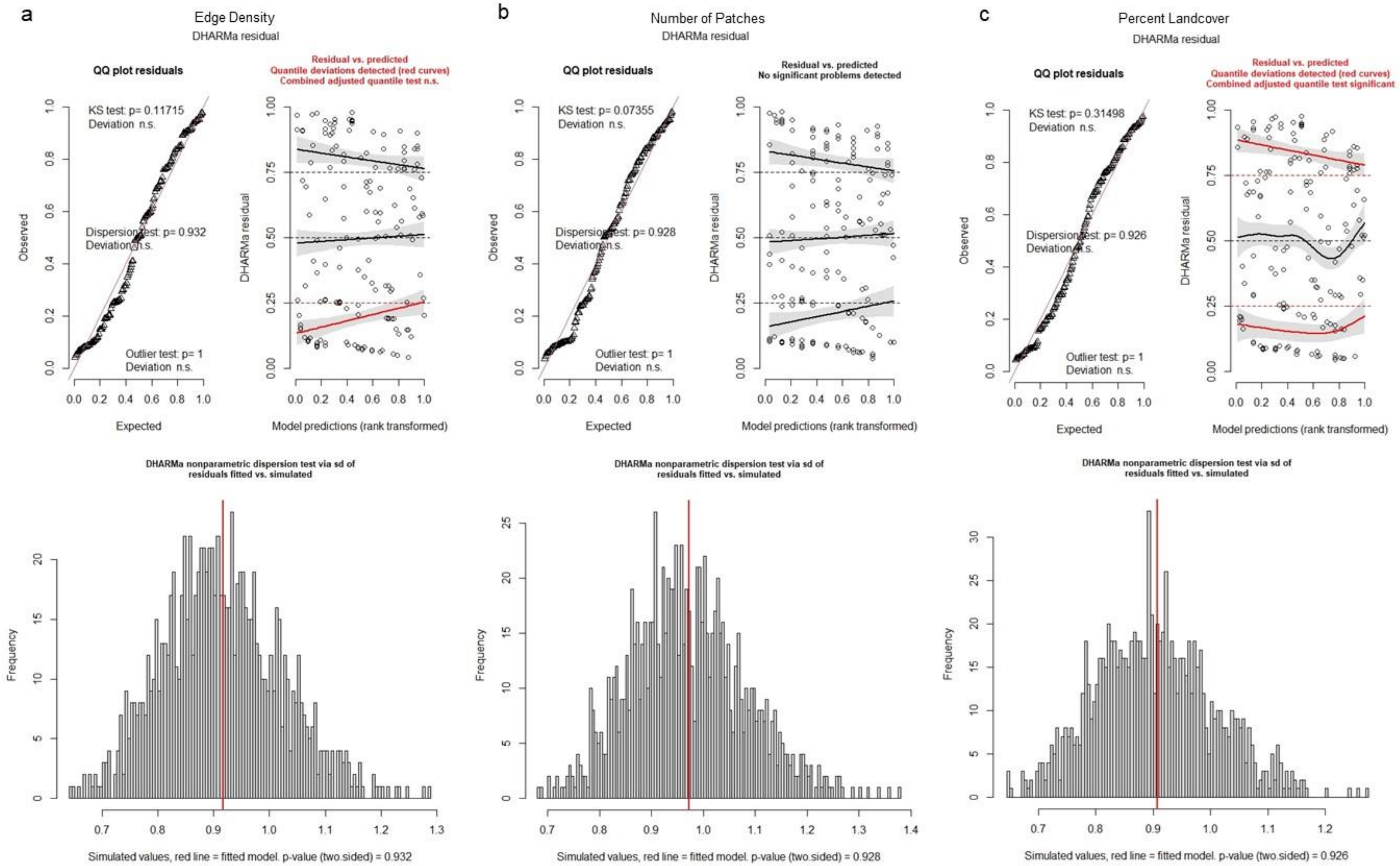

Figure S1: QQ plots, residual diagnostics, and dispersion tests for model fit of most parsimonious model of each candidate set used to predict abundance of *Amblyomma americanum* collected from 157 wild pigs in northern and central Florida. Red line in dispersion test plot indicates the fitted model. a) Edge density, b) Number of patches, c) Percent landcover.

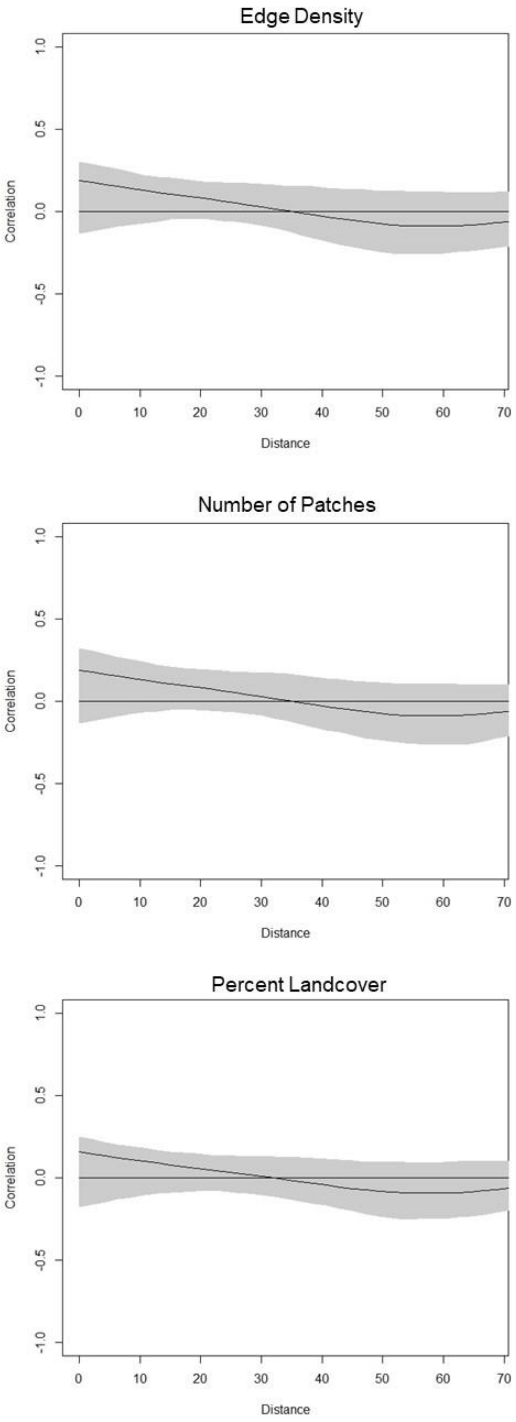

Figure S2: Spatial correlogram plot with 95% confidence intervals to test for residual spatial autocorrelation in each of the three landscape variables used to predict abundance of *Amblyomma americanum* collected from wild pigs in northern and central Florida. 1,000 iterations were used to generate a bootstrap distribution.

Table S1: Variance Inflation Factor (VIF) [*] values for each candidate set of variables within each landscape metric for the prediction of the abundance of *Amblyomma americanum* from wild pigs in northern and central Florida.

	Variance Inflation Factor values for each candidate set		
	Edge Density	Number of Patches	Percent Landcover
Developed open space	1.080651	1.143323	1.043186
Deciduous forest	1.276142	1.632664	1.256606
Mixed forest	1.134104	1.387654	1.092966
Shrub/Scrub	1.528897	1.670567	1.383305
Herbaceous grassland	1.210138	1.243242	1.162314

[*] Comparisons are made between VIF values within each landscape metric to identify the presence of collinearity in the set of variables using a threshold of VIF <3.

Table S2: Akaike Information Criterion (AIC) table of models of three landscape metrics where selected landcover classes[*] are predictor variables to predict the abundance of *Amblyomma americanum* collected from wild pigs in northern and central Florida (2020-2021).

Intercept	Dev. open space	Decid. forest	Mixed forest	Shrub/Scrub	Herb. grassland	df	Log(L_i)[†]	AIC	ΔAIC	AIC_w[‡]
Models of edge density of selected landcover classes										
1.391	X[§]	X	X	0.012437	-0.00929	6	-231.695	475.389	0.000	0.205
1.289	X	X	0.007797	0.013215	-0.00883	7	-230.919	475.839	0.450	0.164
1.181	0.003159	X	0.009587	0.013057	-0.00833	8	-230.223	476.446	1.057	0.121
1.331	0.002213	X	X	0.012201	-0.00901	7	-231.337	476.674	1.285	0.108
1.392	X	-0.00218	X	0.012857	-0.00935	7	-231.661	477.321	1.932	0.078
Models of number of patches of selected landcover classes										
1.316	X	X	X	0.021892	X	5	-236.504	483.008	0.000	0.106
1.465	X	0.019994	X	X	X	5	-236.650	483.301	0.293	0.092
1.111	0.009235	X	0.023128	0.025415	-0.00999	8	-233.907	483.815	0.807	0.071
1.247	0.005662	X	X	0.021306	X	6	-235.920	483.840	0.831	0.070
1.114	0.009175	0.008612	0.018955	0.016897	X	8	-233.990	483.979	0.971	0.065
1.279	0.0095	0.017761	0.014107	X	X	7	-235.089	484.179	1.170	0.059

1.346	X	X	X	0.026004	-0.01029	6	-236.131	484.262	1.254	0.057
1.262	X	X	0.015632	0.026143	-0.00981	7	-235.300	484.599	1.591	0.048
1.265	X	0.008696	0.011459	0.017647	X	7	-235.363	484.725	1.717	0.045
Models of number of patches of selected landcover classes										
1.316	X	X	X	0.021892	X	5	-236.504	483.008	0.000	0.106
1.465	X	0.019994	X	X	X	5	-236.650	483.301	0.293	0.092
1.111	0.009235	X	0.023128	0.025415	-0.00999	8	-233.907	483.815	0.807	0.071
1.247	0.005662	X	X	0.021306	X	6	-235.920	483.840	0.831	0.070
1.114	0.009175	0.008612	0.018955	0.016897	X	8	-233.990	483.979	0.971	0.065
1.279	0.0095	0.017761	0.014107	X	X	7	-235.089	484.179	1.170	0.059
1.346	X	X	X	0.026004	-0.01029	6	-236.131	484.262	1.254	0.057
1.262	X	X	0.015632	0.026143	-0.00981	7	-235.300	484.599	1.591	0.048
1.265	X	0.008696	0.011459	0.017647	X	7	-235.363	484.725	1.717	0.045

[*] Landcover classes are Developed open space (dev. open space), Deciduous forest (decid. forest), Mixed forest, Shrub/Scrub, and Herbaceous grassland (herb. grassland). [†]Log(L_i) is the log likelihood, ΔAIC is the change in AIC values compared to the most parsimonious model within each landscape metric. [‡]AIC_w is the AIC weight, or the conditional probability of the model. [§]Values of X under a variable column for a specific model row indicates that the variable was not included in the model.
